# Determination of epidemiological cut-off values for Narasin, Salinomycin, Lasalocid and Monensin in *Enterococcus faecium*

**DOI:** 10.1093/jac/dkaf113

**Published:** 2025-07-31

**Authors:** Maria Laura Ferrando, Francesca Saluzzo, Rikki Franklin Frederiksen, Jannice Schau Slettemeås, Anne Margrete Urdahl, Roger Simm, Magdalena Skarżyńska, Magdalena Zając, Anna Lalak, Jowita Niczyporuk, Dariusz Wasyl, Léa Duval, Florence Tardy, Claire Schapendonk, Michel Rapallini, Giovanni Tosi, Simona Perulli, Letizia Cirasella, Daniela Maria Cirillo, Mariel G Pikkemaat

**Affiliations:** Division Immunology, Transplant and Infectious Diseases, IRCCS Ospedale San Raffaele (OSR), Via Olgettina 60, Milan 20123, Italy; Division Immunology, Transplant and Infectious Diseases, IRCCS Ospedale San Raffaele (OSR), Via Olgettina 60, Milan 20123, Italy; Department of Animal Health and Food Safety, Norwegian Veterinary Institute (NVI), P.O. Box 64, Ås 1431, Norway; Department of Animal Health and Food Safety, Norwegian Veterinary Institute (NVI), P.O. Box 64, Ås 1431, Norway; Department of Animal Health and Food Safety, Norwegian Veterinary Institute (NVI), P.O. Box 64, Ås 1431, Norway; Department of Biosciences, University of Oslo, P.O. Box 1066, Blindern, Oslo 0316, Norway; Department of Microbiology, National Veterinary Research Institute (PIWet), Partyzantow Avenue 57, Pulawy 24-100, Poland; Department of Microbiology, National Veterinary Research Institute (PIWet), Partyzantow Avenue 57, Pulawy 24-100, Poland; Department of Microbiology, National Veterinary Research Institute (PIWet), Partyzantow Avenue 57, Pulawy 24-100, Poland; Department of Microbiology, National Veterinary Research Institute (PIWet), Partyzantow Avenue 57, Pulawy 24-100, Poland; Department of Microbiology, National Veterinary Research Institute (PIWet), Partyzantow Avenue 57, Pulawy 24-100, Poland; ANSES, Ploufragan, Plouzané Niort laboratory, Mycoplasmology Bacteriology and Antimicrobial Unit (ANSES), Zoopole Les Croix, BP 53, Ploufragan 22440, France; ANSES, Ploufragan, Plouzané Niort laboratory, Mycoplasmology Bacteriology and Antimicrobial Unit (ANSES), Zoopole Les Croix, BP 53, Ploufragan 22440, France; Wageningen University and Research, Wageningen Food Safety Research (WFSR), P.O. Box 230, Wageningen 6708 WB, The Netherlands; Wageningen University and Research, Wageningen Food Safety Research (WFSR), P.O. Box 230, Wageningen 6708 WB, The Netherlands; Istituto Zooprofilattico Sperimentale della Lombardia e dell’Emilia-Romagna (I.Z.S.L.E.R.), Forlì 47122, Italy; Istituto Zooprofilattico Sperimentale della Lombardia e dell’Emilia-Romagna (I.Z.S.L.E.R.), Forlì 47122, Italy; Istituto Zooprofilattico Sperimentale della Lombardia e dell’Emilia-Romagna (I.Z.S.L.E.R.), Forlì 47122, Italy; Division Immunology, Transplant and Infectious Diseases, IRCCS Ospedale San Raffaele (OSR), Via Olgettina 60, Milan 20123, Italy; Wageningen University and Research, Wageningen Food Safety Research (WFSR), P.O. Box 230, Wageningen 6708 WB, The Netherlands

## Abstract

**Objectives:**

To establish epidemiological cut-off values (ECOFFs) for the polyether ionophores narasin, salinomycin, lasalocid and monensin in *Enterococcus faecium*.

**Methods:**

MICs were measured using the broth microdilution method according to ISO 20776-1 (2019). Method validation involved ≥10 replicates of antimicrobial susceptibility testing of *Enterococcus faecalis* ATCC 29212. A total of 182 *E. faecium* isolates from various sources were tested in five European laboratories. The ECOFFinder tool from EUCAST was used to establish the ECOFFs for 122 WT isolates, verified by PCR or WGS.

**Results:**

Method validation showed consistency, with acceptable variation within ±1 2-fold dilution. The ECOFF for narasin was 0.5 mg/L, considerably below the current EUCAST ECOFF for *E. faecium* (ECOFF = 2 mg/L). Salinomycin and lasalocid ECOFFs were 1 and 2 mg/L, respectively. Strains carrying the previously identified *narAB* resistance genes clearly manifested a separate MIC distribution for narasin and salinomycin, but not for lasalocid, although a clear bias to the higher MIC values within the normal distribution could be observed. Monensin apparently displayed a broader MIC range (0.5–64 mg/L) with multiple modes, which precluded the establishment of an ECOFF for monensin.

**Conclusions:**

The study yielded novel ECOFFs for distinguishing WT *E. faecium* strains for the key veterinary ionophores, providing a mainstay for a better understanding of ionophore resistance in enterococci.

## Introduction

Modern intensive broiler farming heavily relies on ionophore coccidiostats in feed to prevent coccidiosis caused by *Eimeria* spp.^1^ Despite their disease-controlling purpose, within the European Economic Area (EEA), ionophores (in particular narasin, salinomycin, lasalocid, monensin, maduramicin and semduramicin) are considered feed additives, which implies that they are regulated under EC 1831/2003.^2^ As a consequence, consumption statistics are lacking, but sporadic data indicate their use largely exceeds that of therapeutic veterinary antibiotics in poultry. UK data show that in 2019, a total of 265 tons of ionophores were used, versus 20–30 tons of medically important antibiotics (MIAs) in poultry, whereas in Finland in 2020 total use of ionophores was 20.8 tons, versus 8.9 tons of MIAs used in all species.^3^ Outside the EEA their use may even be more extensive, considering their application as antimicrobial growth promoters in all major production species. Notably, the polyether ionophores exhibit not only antiprotozoal activity but they are also effective against Gram-positive bacteria, including species in the *Enterococcus* genus relevant to veterinary and human medicine. *Enterococcus faecium*, a commensal in the human and animal gastrointestinal tract, has emerged as a significant nosocomial pathogen, often resistant to a broad spectrum of antimicrobial drugs.^4,5^ In particular, the rapid and continuous increase of VAN resistance, a last resort antibiotic for the treatment of Gram-positive highly resistant infections, is of great concern.^6^ Antimicrobial resistance genes in *E. faecium* are often located on mobile genetic elements.^7,8^ The recent discovery of a plasmid-encoded ABC-type transporter conferring resistance against the ionophores narasin, salinomycin and maduramicin,^9^ puts the massive use of ionophores in a new perspective. These resistance genes (*narAB*) were found to be co-located on plasmids with genes conferring resistance against MIAs^9,10^ including VAN, which suggests that an ionophore driven co-selection may occur.

There is very limited information on ionophore susceptibility in enterococci, and interpretation of monitoring data is hampered by the fact that Epidemiological Cut-Off values (ECOFFs)^11^ are virtually lacking. Historically, cut-off values of >2 mg/L for narasin and >8 mg/L or even >16 mg/L for salinomycin were used.^12,13^ Previously, a Tentative ECOFF (TECOFF) for narasin was initially suggested at an MIC value of >4 mg/L. This TECOFF was adjusted after Nilsson *et al*.^14,15^ analysed *E. faecium* strains isolated from broilers with a putative narasin resistance mechanism, and proposed a TECOFF for narasin ≥2 mg/L, which is the value currently recorded in the EUCAST database. An ECOFF study, taking into account the recently discovered *narAB* ionophore resistance determinants, could clarify susceptibility profiles in *E. faecium*.

In our study, we analysed MIC distributions for narasin, salinomycin, lasalocid and monensin in 122 WT *E. faecium* strains originating from humans, animals and food, as well as 60 strains carrying the *narAB* genes. Results were generated in five laboratories across various European countries, and we propose new ECOFFs for narasin, salinomycin and lasalocid. A deviating MIC distribution was observed for monensin, which precludes establishing an ECOFF.

## Materials and methods

### Ethics approval

All procedures involving the collection and handling of bacterial isolates were conducted according to the ethical standards of the respective institutions and national and international guidelines on microbiological research.

### Collection of *E. faecium* isolates

*E. faecium* isolates were collected between 2005 and 2023 from five European Research Centres: (1) IRCCS Ospedale San Raffaele (OSR), Italy (Lab 1); (2) Norwegian Veterinary Institute (NVI), Norway (Lab 2); (3) Wageningen Food Safety Research, Wageningen University and Research (WFSR), the Netherlands (Lab 3); (4) National Veterinary Research Institute (PIWet), Poland (Lab 4); and (5) Agence Nationale de Sécurité Sanitaire de l’Alimentation (ANSES), France (Lab 5). For this study a total of 182 *E. faecium* strains were collected, comprising 122 WT strains (presumably narasin/salinomycin^S^, lacking the *narAB* genes), and 60 strains presumably resistant to narasin and salinomycin (narasin/salinomycin^R^, due to the presence of the *narAB* genes) (Table S1, available as Supplementary data at *JAC* Online). Strains originated from different sources, including poultry (*n* = 128), human (*n* = 50) and others (*n* = 4) (see Table S2 for metadata and MICs, available as Supplementary data at JAC Online). All isolates were characterized by MALDI-TOF MS (Vitek MS, bioMérieux, France) and/or by WGS.

### MIC determination by broth microdilution

Stock solutions of ionophores were prepared by Lab 3 dissolving the standard powder in pure methanol with the following concentrations: 1.0 mg/mL for narasin (1457458 USP, Sigma-Aldrich), salinomycin (sc-236851, Santa Cruz Biotechnology) and monensin (1445481 USP2, Sigma-Aldrich) and 0.2 mg/mL for lasalocid (sc-362029A, Santa Cruz Biotechnology). The concentration range tested was 0.03–32 mg/L for narasin, salinomycin and lasalocid and 0.06–64 mg/L for monensin. The MICs of the ionophores were determined using the broth microdilution (BMD) according to ISO 20776-1, 2019 guidelines and by EUCAST standard operating procedure, EUCAST SOP 10.2 (2 December 2021).^16^ Briefly, each strain was grown at 37°C for 18 ± 2 h on Columbia Blood Agar, and several (3–4) colonies were resuspended in 5 mL of sterile demineralized water (Thermo Fisher, code T3339) to achieve a turbidity of 0.5 McFarland. Successively the 0.5 McFarland of bacterial suspension was diluted 1:100 in Sensititre^®^ CAMHBT (Thermo Fisher, code YT3462). The final inoculum concentration was ∼5 × 10^5^ cfu/mL. The plates were incubated at 35 ± 1°C for 18 ± 2 h. MICs were determined as the lowest concentration of the agent that inhibited visible growth. Due to the bacteriostatic properties of the ionophores, pinpoint growth was occasionally observed.^17^ In consultation with EUCAST, a reference document was prepared according to which all participants conducted the MIC assessment (Figure S1).

### Validation of the antimicrobial susceptibility test method

*E. faecalis* ATCC 29212 was used as quality control strain (QC)^18^ for the antimicrobial susceptibility test (AST). The validation was conducted by testing the QC with ≥10 AST in triplicate on different days for each ionophore. The variation between laboratories was considered acceptable if the modes of the QC distributions were equal to or within one 2-fold dilution of the most frequently observed mode.^19^ Additionally, a blind ring trial was organized from Lab 3 comprising five *E. faecium* and five *E. faecalis* strains.

### *narAB* gene detection by WGS or PCR

Bacterial DNA was extracted using an automated DNA extraction platform with some variation in each lab. Briefly, samples were sub-cultured in Middlebrook 7H9 broth to perform DNA extraction using either Maxwell 16 Cell DNA Purification kit (Promega) or QIAamp DNA Mini Kit (Qiagen, Germany). Illumina technology was used for a paired-end (2 × 150 bp) run on the NextSeq 500 next-generation sequencing platform (San Diego, CA, USA) following the manufacturer’s instructions. Sequencing data were pre-processed by trimming Illumina adapters.^20^ Trimmed reads were assembled using Unicycler software (https://github.com/rrwick/Unicycler). The *narAB* sequence (GenBank: MN590310.1)^9^ was detected in assembled contigs using Abricate software (https://github.com/tseemann/abricate) with a custom database containing the reference sequences for the genes. Sequences with at least 95% identity to *narAB* were accepted.

PCR was performed according to a previously published method^21^ using the following primers: 5′-TGTTCCTGGGGATGTTGCTC-3′ and 5′-AGAGCGTCGCAAGTTTCTCA-3′ for *narA* (ABC ATPase gene) and 5′-AGCTGCGTATGGCTCCATTT-3′ and 5′-GCTGATGCTAAGCCAATGCC-3′ for *narB* (ABC permease gene).

### Data analysis

ECOFFs for the ionophores were computed using ECOFFinder software (previously available at www.eucast.org/mic_and_zone_distributions_and_ecoffs)^11,22^ and through visual analysis of the aggregated dataset. WT upper limits were determined statistically at 95%, 97.5% and 99% of the modelled population. MIC distributions qualified for aggregation when they exhibited unimodality and the most frequent MIC in valid distributions fell within ±1 dilution of the modal MIC, following EUCAST guidelines (EUCAST, SOP 10.2.)^23^

## Results

### Validation of the BMD method

Validation of the method involved two phases. The first phase included repetitive AST for each ionophore with the reference strain *E. faecalis* ATCC 29212 (QC). The second phase comprised blind AST on five *E. faecium* and five *E. faecalis* strains. Table 1 and Figure S2 present MICs of the five laboratories testing the QC strain 10 times in triplicate. The MICs of narasin ranged from 0.06 to 0.5 mg/L, with the highest proportions at 0.125 (39%) and 0.25 mg/L (50%), suggesting a potential modal MIC value between these concentrations.

**Table 1.** Phase 1 validation test for the AST of the QC: MICs for four ionophores (NAR, SAL, LAS and MON) of the reference strain *E. faecalis* ATCC 29212 (QC)

	NAR (mg/L)					Modal MIC	Total MICs	SAL (mg/L)					Modal MIC	Total MICs	LAS (mg/L)					Modal MIC	Total MICs	MON (mg/L)					Modal MIC	Total MICs
	0.06	0.125	0.25	0.5	1			0.125	0.25	0.5	1	2			0.125	0.25	0.5	1	2			2	4	8	16	32		
Lab 1		4	**24**	2		0.25	30		3	**25**	2		0.5	30			**23**	7		0.5	30		**17**	13			4	30
Lab 2	*9*	**14**	7			0.125	30		15	**16**			0.5	31		3	**17**	10		0.5	30		**19**	6	*6*		4	31
Lab 3		13	**16**	1		0.25	30		8	**21**	1		0.5	30			**26**	4		0.5	30	3	**17**	10			4	30
Lab 4		6	**22**	2		0.25	30		1	**23**	9		0.5	33			12	**18**		1	30	7	**23**				4	30
Lab 5	*3*	**21**	6			0.125	30	*3*	**15**	13	1		0.25	32		1	**18**	12		0.5	31	4	5	**11**	*10*		8	30
All labs (n)	*12*	58	**75**	5		**0**.**25**	150	*3*	42	**98**	13		**0**.**5**	156		4	**96**	51		**0**.**5**	151	14	**81**	40	*16*		**4**	151
All labs (%)	*8%*	39%	**50%**	3%				*2%*	27%	**63%**	8%					3%	**64%**	34%				9%	**54%**	26%	*11%*			

The most frequent MIC is in bold, values outside the acceptable range are in italics.

Regarding salinomycin, MIC values varied from 0.125 to 1 mg/L, with 63% of the dataset exhibiting an MIC of 0.5 mg/L. Lasalocid displayed MICs ranging from 0.25 to 1 mg/L, with 63% of the results showing an MIC of 0.5 mg/L. For monensin, observed MIC values ranged from 2 to 16 mg/L. The results generated by Lab 1–4 showed a clear mode at 4 mg/L (54%). The MIC distribution of Lab 5 showed a somewhat broader range and did not yield the expected normal shape. Overall, the modal MICs of the QC for narasin, salinomycin and lasalocid varied within one 2-fold dilution, which is considered the acceptable technical variation for the BMD method.^16,19^ For monensin, a wider variability was observed, but the results were deemed acceptable for proceeding to the next phase.

To assess the accuracy of our BMD method, the second phase of the validation involved blind testing of 10 strains (five *E. faecium* and five *E. faecalis*) originating from Lab 3’s collection and referred to as ICONIC 1–10. Strains were selected based on preliminary salinomycin AST results only known to the organizer. The majority of the laboratories obtained similar conclusions regarding the susceptibility of the strains to narasin and salinomycin (Table S3), identifying four strains with a narasin MIC ≤ 0.5 mg/L and a salinomycin MIC ≤ 1 mg/L (ICONIC 2, ICONIC 3, ICONIC 4 and ICONIC 7) and six strains with a narasin MIC > 0.5 mg/L and a salinomycin MIC > 1 mg/L (ICONIC 1, ICONIC 5, ICONIC 6, ICONIC 8, ICONIC 9 and ICONIC 10). Lab 5 obtained slightly deviating salinomycin MICs for ICONIC 5 and ICONIC 7. Results for lasalocid were also consistent across the different laboratories. Results for monensin showed more variation among the laboratories. For 7 out of 10 strains, the MIC values spanned 3–4 2-fold dilutions, underscoring the precariousness of the assay for monensin.

### MIC distributions and ECOFF estimations in WT *E. faecium* strains

A comprehensive study was carried out on 122 unique putatively WT *E. faecium* strains, not carrying the *narAB* genes, across five laboratories to determine the ECOFFs for the four ionophores.

**Figure 1.**
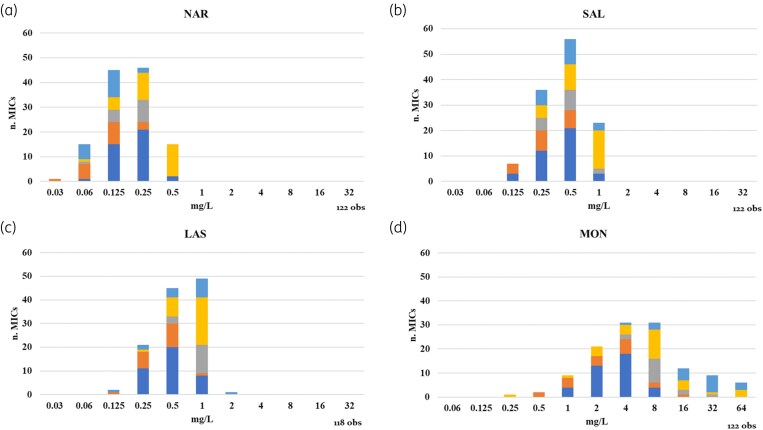
Combined MIC distributions for the ionophores resulting from the analysis of *E. faecium* isolates lacking the resistance genes narAB (NAR/SAL^S^), generated by five different research laboratories (single distributions are provided in Table S3). (a) narasin (NAR), (b) salinomycin (SAL), (c) lasalocid (LAS) and (d) monensin (MON). Lab 1, OSR; Lab 2, NVI; Lab 3, WFSR; Lab 4, PIWet; and Lab 5, ANSES. Obs, observations: number of isolates tested in AST.

**Figure 2.**
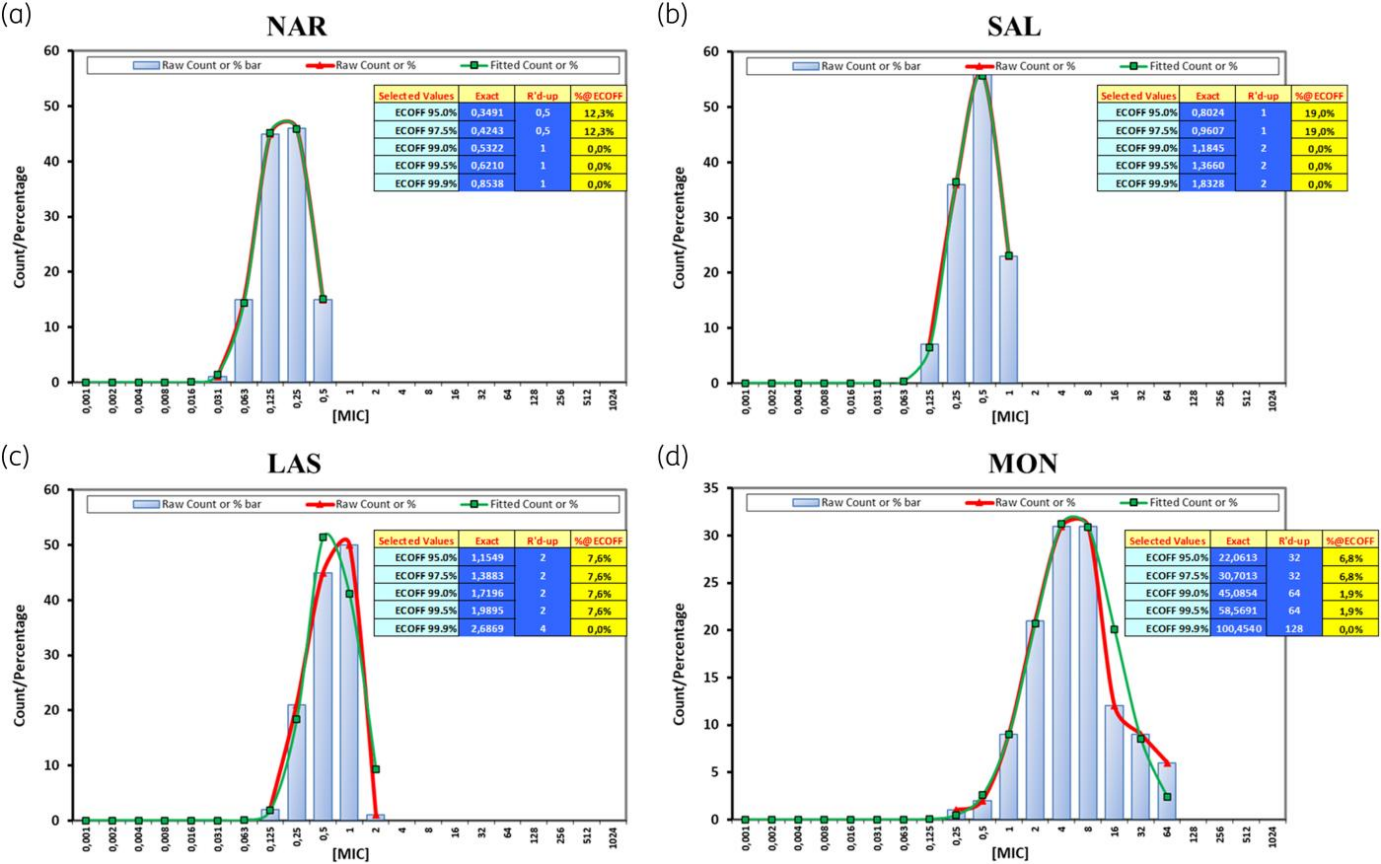
ECOFFinder results (aggregated data from five laboratories) for each ionophore (a) narasin (NAR), (b) salinomycin (SAL), (c) lasalocid (LAS) and (d) monensin (MON) MIC distributions of *E. faecium* lacking the resistance genes narAB (NAR/SAL^S^). The graphs present the raw data using both column and curve formats (thick red curve), alongside the estimated best-fit curve for the WT population (in green with squares, partially overlapping). The table details a range of ECOFFs selected based on the percentage of the WT. It includes the ‘exact’ ECOFF, the ECOFF rounded up to the next 2-fold dilution, and the percentage of isolates in the raw data that exceed this rounded-up ECOFF, denoted as %@ECOFF, indicating the proportion of population isolates sitting at these ECOFF values.

According to EUCAST SOP 10.2 section 4.1,^16^ the ECOFF should be calculated as the mean of the values calculated for each individual laboratory. However, some of the individual lab distributions appeared truncated at the upper end (Table S4) which may have been caused by the fact that exclusively genetically WT (lacking *narAB* gene) isolates were included. This precluded the use of ECOFFinder on individual distributions. The combined MIC distribution for narasin exhibited a Gaussian distribution, with two modal MICs at 0.125 (36.9% of the MICs) and 0.25 mg/L (37.7% of the MICs) (Figure 1a, Table S4). The modal MICs for individual laboratories differed at most ±1 2-fold dilution from the most frequent two modes (0.125–0.25 mg/L) (Table S4). Using the ECOFFinder tool, the ECOFF for 97.5% of the WT population was established at 0.5 mg/L. All tested isolates had an MIC at/or below 0.5 mg/L for narasin (% @ECOFF = 0.0%) (Figure 2a).

The aggregated MIC distribution for salinomycin ranged from 0.125 to 1 mg/L, with a mode of 0.5 mg/L (45.4% of the MICs) (Figure 1b, Table S4). Three laboratories generated an MIC distribution with a modal MIC of 0.5 mg/L, while Lab 2 and Lab 4 reported a modal MIC of 0.125 and 1 mg/L, respectively. The ECOFF for 97.5% of the WT population was calculated at 1 mg/L using the ECOFFinder tool. None of the analysed strains exceeded this MIC of 1 mg/L for salinomycin (Figure 2b).

Although a previous study indicated the absence of cross-resistance between *narAB* based narasin and salinomycin resistance and the other polyether ionophores lasalocid and monensin,^9^ the analysis of the MIC distributions for lasalocid and monensin was also based on the strains lacking *narAB.* The combined MIC distribution for lasalocid ranged from 0.125 to 2 mg/L, with a mode of 1 mg/L (42% of the MICs) (Figure 1c, Table S4). The ECOFFs for 97.5% of the WT population was set at 2 mg/L using the ECOFFinder tool. None of the strains exceeded the MIC of 2 mg/L for lasalocid (Figure 2c).

As already observed during the validation, monensin showed the greatest inter- and intra-laboratory MIC variation, ranging from 0.25 to 64 mg/L with a mode between 4 and 8 mg/L with a non-log-normal distribution (Figure 1d, Table S4). An explanation for this variation is currently not known. Due to the challenges experienced with determining consistent MICs of monensin, the wide distribution of MICs, and the observed intra-laboratory variation, it was not possible to establish a reliable ECOFF for monensin.

### MIC distributions including narAB-positive strains

**Figure 3.**
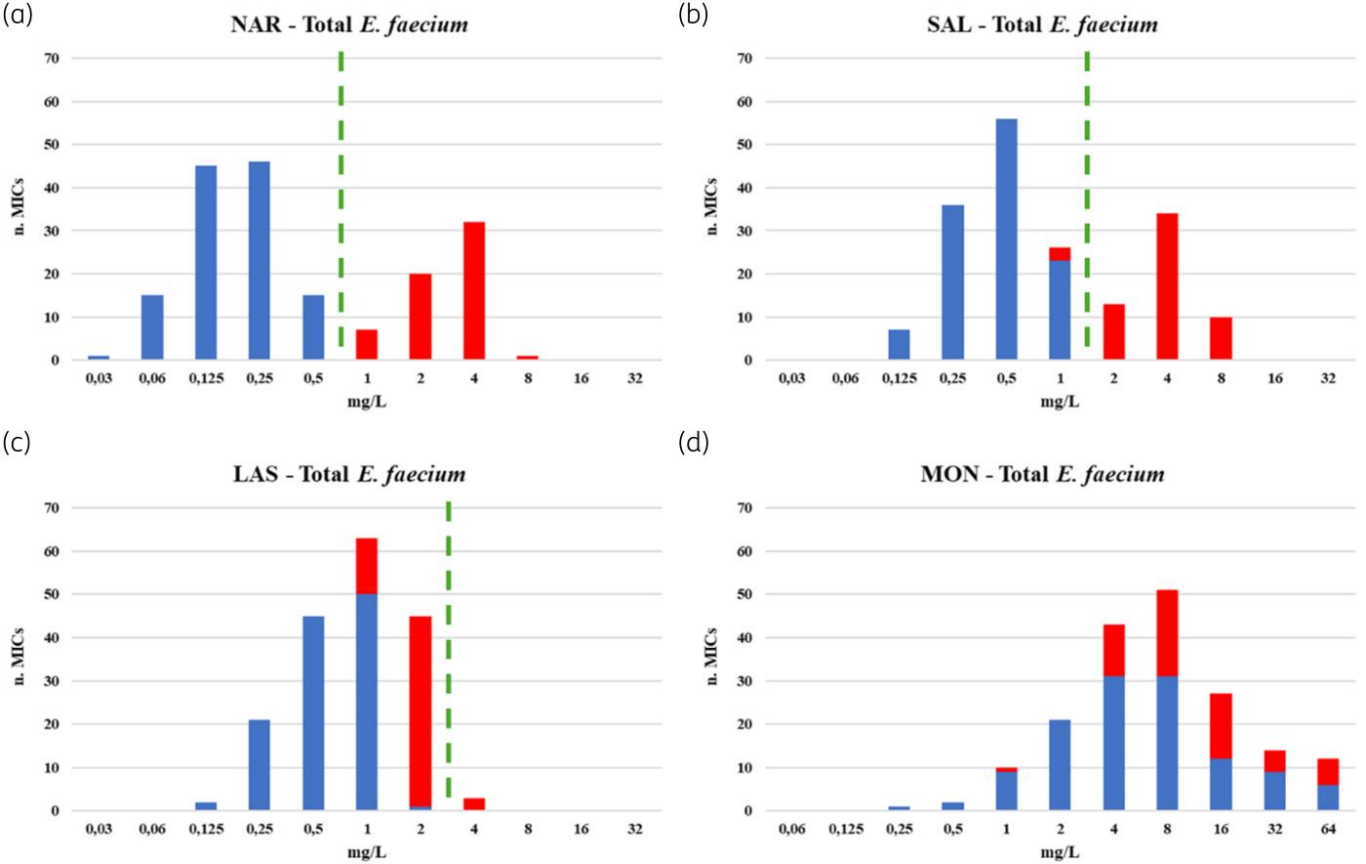
MIC distributions for all *E. faecium* against ionophores. (a) NAR, (b) SAL, (c) LAS within the test range of 0.03–32 mg/L and (d) MON in the range of 0.06–64 mg/L. The dotted line represents the proposed ECOFFs for NAR, SAL and LAS. The colour of the columns indicates whether the isolates carried the resistance genes narAB (in red) identified by WGS or PCR or not (WT isolates; in blue). Obs, observations: number of isolates tested in AST.

Figure 3 shows the MIC population distributions for narasin, salinomycin, lasalocid and monensin including 60 supplementary isolates carrying the *narAB* genes. When tested for narasin susceptibility, the *narAB-*positive strains revealed a distinct distribution separated from the WT population at the established ECOFF, with MICs ranging from 1 to 8 mg/L with a modal MIC of 4 mg/L (Figure 3a, distributions per individual lab in Table S4). For salinomycin, a similar profile was obtained. The *narAB*-positive strains showed a separate distribution ranging from 2 to 8 mg/L, also with a modal MIC of 4 mg/L (Figure 3b). The MIC distribution showed an almost clear separation between WT and *narAB*-positive isolates, except for three strains from Lab 5 that appeared to harbour *narAB* and displayed an MIC for salinomycin of 1 mg/L. All the remaining *narAB*-positive strains showed an MIC of >1 mg/L. In conclusion, a ≥95% correlation between phenotypic and genotypic resistance was observed for narasin and salinomycin.

Interestingly, although *narAB*-related cross-resistance was not anticipated for lasalocid, the MICs of the *narAB*-positive strains indicate a correlation between the presence of the genes and reduced susceptibility to lasalocid. When combining WT and *narAB*-positive strain distributions, a broader MIC histogram emerged, ranging from 0.25 to 4 mg/L, with a mode of 1 mg/L (Figure 3c), in which all *narAB*-positive isolates display an MIC > 0.5 mg/L and are positioned at the upper end of the distribution. Finally, for monensin, the combined MIC distribution for WT and narAB-positive strains more or less resembled that of the WT strains, indicating that the presence of *narAB* does not influence monensin susceptibility. Nevertheless, it should be noted that Lab 1’s data, comprising WT isolates exclusively derived from humans and *narAB*-positive isolates originating from poultry exposed to monensin, showed a distinct separation between the two populations (Table S4), indicating at least some adaptation towards monensin may be occurring in the field.

## Discussion

Concerns have been raised regarding the development of antimicrobial resistance and the potential transfer of resistance against antibiotics used in both human and veterinary medicine.^24^ Polyether ionophores, a class of antibiotics widely used as feed additives in production animals for their coccidiostat properties, have also been shown to be effective against Gram-positive bacteria.^25–27^ They are not used in human medicine, but some recent studies highlight the risk of ionophore use promoting the dissemination of bacterial resistance against MIAs.^14,21,28^ Also it cannot be excluded that they can have a direct effect on the pathogenicity of bacteria, as it was recently shown that reduced susceptibility to monensin in *Staphylococcus aureus* was associated with enhanced virulence.^29^ Although *E. faecium* and, in particular VRE, is considered a high-priority pathogen,^30^ AST of enterococci in the animal domain has received decreasing attention because of its voluntary status under prevailing EU legislation.^31^ Monitoring data are scarce and the recommended AST panel for enterococci does not include a representative of the polyether ionophores.^27^ The only recent systematically collected results originate from Norway, where a decrease in the occurrence of narasin resistance was detected in *E. faecium* from 24.7% in 2018 to 15.6% in 2020 (at the applied cut-off of MIC > 2 mg/L),^32^ as a result of phasing out narasin as coccidiostat for broilers, since 2015. From historical national monitoring data originating from Denmark, Sweden and the Netherlands, a significant prevalence of resistance against narasin or salinomycin can be deduced.^32–34^ In 2014, resistance rates for narasin in *E. faecium* isolated from broilers reported from Sweden were 77% (at a cut-off of >2 mg/L), whereas no narasin-resistant isolates were observed from other production animals. For *E. faecalis* the rates were lower, but still considerably high at 41%.^33^ The last available data from the Netherlands (2013) show that at the time 53.3% of *E. faecalis* and 76.8% of *E. faecium* isolates from broilers displayed a salinomycin MIC > 2 mg/L.^35^ Danish data interpretation is somewhat hampered by the fact that the lowest tested concentration of salinomycin was 2 mg/L, nevertheless, 84.8% of Danish *E. faecium* isolates from broiler meat displayed a salinomycin MIC > 2 mg/L.^34^ For *E. faecalis*, this number was considerably lower at 17.9%. Even though methods may not have been fully harmonized, and different TECOFFs or presumptive breakpoints were applied to determine resistance rates before this publication, past literature clearly demonstrates that the prevalence of ionophore resistance is high in enterococci from broilers.

Considering the connection existing between animal and human health (One Health approach) and the possible role of ionophores in the dissemination of resistance against MIAs, the determination of ECOFFs for these ionophores is of relevance for monitoring and managing antimicrobial resistance in Gram-positive bacteria of both human and veterinary importance.

Nilsson *et al.*  ^14^ suggested an ECOFF for narasin between 1 and 2 mg/L, which is in line with the current EUCAST ECOFF of >2. For salinomycin Dutch and Danish surveillance programs historically applied a cut-off of >4 mg/L. According to our data, ECOFFs could be established at 0.5 mg/L for narasin and 1 mg/L for salinomycin, which is lower than the historically applied cut-off values, implying resistance rates could be even higher than reported. It cannot be excluded that these differences have a methodological origin. The historical surveillance data (which make up the vast majority of the EUCAST data) have been obtained from freeze-dried panels, while the current AST assay was prepared from −80°C stock solutions. Comparison of current and historically obtained MICs for the same isolates (available for Lab 2 and Lab 3); however, did not indicate a structural deviation. Another factor might be the judgement of bacterial growth in the panels. Occasionally pinpoint growth was observed, which was (upon consultation with EUCAST) ignored. Notably, in contrast to the EUCAST data, the current set of isolates that was used to determine the narasin and salinomycin ECOFFs exclusively consisted of WT.

The ECOFF for lasalocid was established at 2 mg/L. Although no bimodal distribution of MIC values was observed after including the *narAB*-positive strains, the latter consistently showed slightly elevated MICs, indicating some form of reduced susceptibility associated with the presence of the genes. However, since no secondary MIC distribution emerged from the analysis, we did not find indications for the existence of lasalocid -specific resistance mechanisms. The only literature data on lasalocid susceptibility of enterococci originate from a study by Butaye *et al.*^36^ who tested 24 *E. faecium* and 21 *E. faecalis* poultry isolates and found an unimodal distribution with a similar mode of 1 mg/L for both species.

For monensin significant variation in MIC distributions was noted, both inter- and intra-laboratory. In addition, sometimes a pattern that resembled the ‘Eagle effect’, a paradoxical phenomenon in which bacterial survival is observed at concentrations exceeding the MIC, was observed.^37^ However, it cannot be excluded that the observed variability reflects solubility issues, as it is known that monensin has poor water solubility. Due to the observed variation, it is not possible to establish a (T)ECOFF for monensin. Remarkably, one population of poultry strains, which was known to originate from broilers exposed to monensin during their life cycle, exhibited MIC values consistently higher than 16 mg/L, suggesting the development of reduced susceptibility to this ionophore. Reduced susceptibility to monensin has been attributed to a transient phenomenon of reversible adaptation, due to a temporary increase of the bacterial cell wall in the presence of high concentrations of the ionophore.^38^ More recent research however showed that reduced susceptibility in *S. aureus* is associated with mutational changes.^29^ This raises the question of what the underlying mechanism for reduced monensin susceptibility might be in enterococci.

The use of ionophores continues to be widespread in poultry due to their effectiveness against coccidiosis, although vaccines do constitute a feasible alternative.^1,39,40^ This comprehensive study sheds light on the susceptibility patterns of *E. faecium* to various ionophores and will contribute to standardized monitoring and surveillance. Moreover, it underlines the importance of understanding both phenotypic and genotypic aspects of antimicrobial resistance. The fact that narasin and salinomycin resistance is occurring at a much higher incidence than previously thought, combined with the observations of its association with resistance against MIAs may have significant implications for the use of ionophores as feed additives. The results also call for more intensive antimicrobial resistance monitoring of enterococci in the animal domain and emphasize the need to look beyond the MIAs.

## Supplementary Material

dkaf113_Supplementary_Data

## Supplementary data

Figures S1 and S2 and Tables S1–S4 are available as Supplementary data at *JAC* Online.
