# Supplementary material for: Determination of epidemiological cut-off values for Narasin, Salinomycin, Lasalocid and Monensin in Enterococcus faecium
Source: J Antimicrob Chemother. 2025 Jul 31;80(9):2361–8. doi: 10.1093/jac/dkaf113 (PMC12404726; doi:10.1093/jac/dkaf113)
Supplement: dkaf113_Supplementary_Data [file dkaf113_supplementary_data.zip › Supplementary Figures (figures S1 S2)_rev.docx]

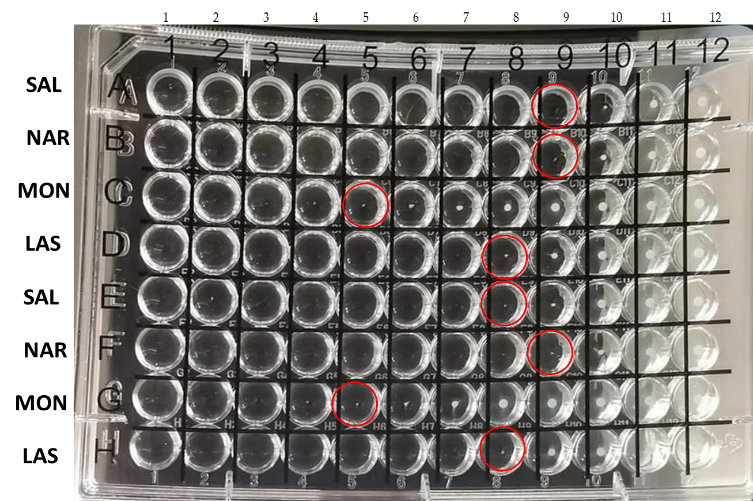

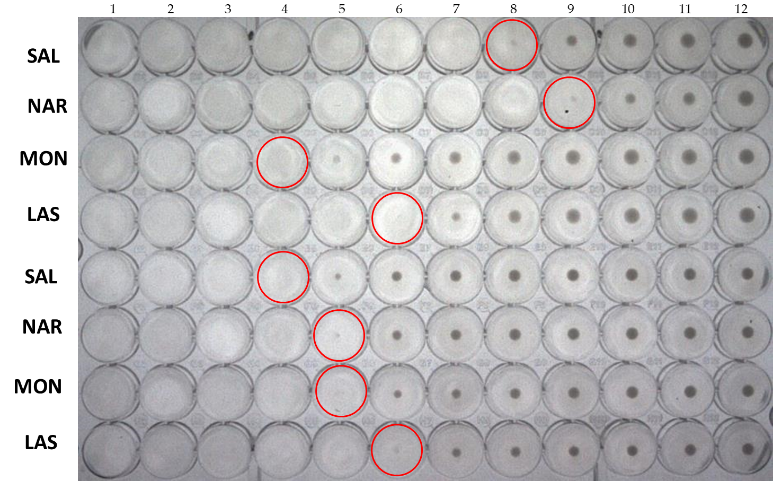


*
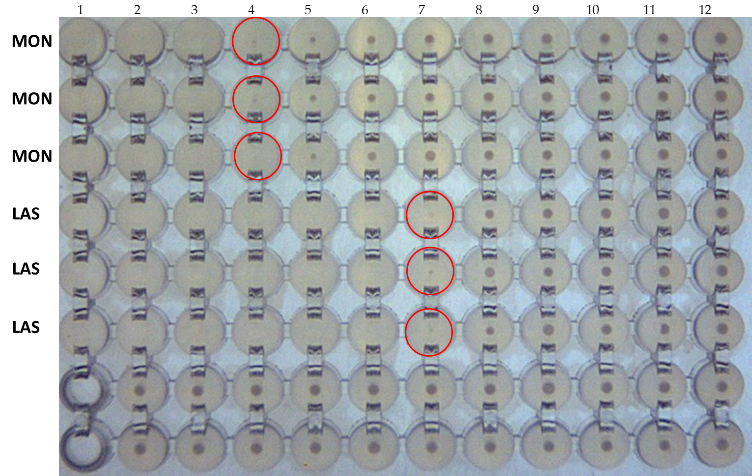
*

***Figures S1****: Two examples of determining MIC endpoints for the ionophores SAL, NAR, MON, and LAS when testing E. faecium strains. The red circle indicates the correct MIC determination.*

***Figure S2****: MIC distributions of the reference strain E. faecalis ATCC 29212 (QC) against ionophores A) NAR, B) SAL, C) LAS within the test range of 0.03–32 mg/L, and D) MON in the range of 0.06–64 mg/L. Each of the five laboratories tested the QC in triplicates ten times. Obs=* *observations: number of isolates tested in A*
