# Supplementary material for: Determination of epidemiological cut-off values for Narasin, Salinomycin, Lasalocid and Monensin in Enterococcus faecium
Source: J Antimicrob Chemother. 2025 Jul 31;80(9):2361–8. doi: 10.1093/jac/dkaf113 (PMC12404726; doi:10.1093/jac/dkaf113)
Supplement: dkaf113_Supplementary_Data [file dkaf113_supplementary_data.zip › Supplementary_Tables_(Table_S1_S3_S4)_revised.docx]

|  | **WT (NAR/SAL^S^)** | **NAR/SAL^R^** | **Total** |
| --- | --- | --- | --- |
| **Lab 1** | 39 | 20 | 59 |
| **Lab 2** | 19 | - | 19 |
| **Lab 3** | 15 | 11 | 26 |
| **Lab 4** | 30 | 10 | 40 |
| **Lab 5** | 19 | 19 | 38 |
| **Total** | **122** | **60** | **182** |

***Table S1****: Number of* E. faecium *isolates provided from each laboratory for the study. Susceptible WT strains to NAR and SAL (NAR/SAL^S^), resistant strains to NAR and SAL (NAR/SAL^R^). Lab 1=OSR, Lab 2=NVI, Lab 3=WFSR, Lab 4=PIWet and Lab 5=ANSES.*

|  | **Lab 1** | **Lab 2** | **Lab 3** | **Lab 4** | **Lab 5** | range (mg/L) |
| --- | --- | --- | --- | --- | --- | --- |
|  | **NAR** mg/L | | | | |  |
| **ICONIC 1** | 2 | 4 | 4 | 4 | 4 | 2-4 |
| ICONIC 2 | 0.5 | 0.5 | 0.5 | 0.5 | 0.5 | 0.5 |
| ICONIC 3 | 0.125 | 0.25 | 0.125 | 0.125 | 0.06 | 0.06-0.25 |
| ICONIC 4 | 0.25 | 0.25 | 0.125 | 0.125 | 0.06 | 0.06-0.25 |
| **ICONIC 5** | 4 | 4 | 4 | 4 | 2 | 2-4 |
| **ICONIC 6** | 2 | 2 | 2 | 2 | 1 | 1-2 |
| ICONIC 7 | 0.125 | 0.25 | 0.06 | 0.125 | 0.03 | 0.03-0.25 |
| **ICONIC 8** | 2 | 2 | 2 | 4 | 2 | 2-4 |
| **ICONIC 9** | 2 | 2 | 2 | 2 | 1 | 1-2 |
| **ICONIC 10** | 2 | 2 | 2 | 2 | 1 | 1-2 |
|  | **SAL** (mg/L) | | | | | |
| **ICONIC 1** | 4 | 4 | 8 | 8 | 4 | 4-8 |
| ICONIC 2 | 1 | 1 | 1 | 1 | 1 | 1 |
| ICONIC 3 | 0.25 | 0.5 | 0.5 | 0.5 | 0.25 | 0.25-0.5 |
| ICONIC 4 | 0.5 | 0.5 | 0.5 | 0.5 | 0.25 | 0.25-0.5 |
| **ICONIC 5** | 4 | 4 | 8 | 8 | 1 | 1-8 |
| **ICONIC 6** | 4 | 2 | 2 | 2 | 2 | 2-4 |
| ICONIC 7 | 0.25 | 0.25 | 0.25 | 0.25 | 0.06 | 0.06-0.25 |
| **ICONIC 8** | 4 | 4 | 4 | 4 | 2 | 2-4 |
| **ICONIC 9** | 4 | 4 | 2 | 4 | 2 | 2-4 |
| **ICONIC 10** | 4 | 2 | 2 | 2 | 0.5 | 0.5-4 |
|  | **LAS** (mg/L) | | | | | |
| **ICONIC 1** | 2 | 2 | 2 | 2 | 2 | 2 |
| ICONIC 2 | 1 | 2 | 1 | 1 | 2 | 1-2 |
| ICONIC 3 | 0.5 | 0.5 | 0.5 | 0.5 | 0.5 | 0.5 |
| ICONIC 4 | 0.5 | 1 | 0.5 | 1 | 0.5 | 0.5-1 |
| **ICONIC 5** | 2 | 2 | 2 | 2 | 2 | 2 |
| **ICONIC 6** | 2 | 1 | 1 | 1 | 1 | 1-2 |
| ICONIC 7 | 0.25 | 0.5 | 0.125 | 0.25 | 0.25 | 0.125-0.5 |
| **ICONIC 8** | 2 | 2 | 1 | 2 | 2 | 1-2 |
| **ICONIC 9** | 2 | 2 | 1 | 1 | 1 | 1-2 |
| **ICONIC 10** | 2 | 2 | 1 | 2 | 1 | 1-2 |
|  | **MON** (mg/L) | | | | | |
| **ICONIC 1** | 8 | 8 | 8 | 16 | 32 | 8-32 |
| ICONIC 2 | 64 | > 64 | 8 | 64 | 64 | 8->64 |
| ICONIC 3 | 2 | 2 | 2 | 2 | 0.25 | 0.25-2 |
| ICONIC 4 | 4 | 4 | 4 | 4 | 4 | 4 |
| **ICONIC 5** | 16 | > 64 | 8 | 64 | 16 | 8->64 |
| **ICONIC 6** | 8 | 4 | 4 | 4 | 2 | 2-8 |
| ICONIC 7 | 1 | 4 | 1 | 2 | 0.5 | 0.5-4 |
| **ICONIC 8** | 8 | 8 | 4 | 4 | 16 | 4-16 |
| **ICONIC 9** | 8 | 8 | 8 | 8 | 8 | 8 |
| **ICONIC 10** | 8 | 8 | 8 | 8 | 4 | 4-8 |

***Table S3****. Phase 2 validation of the AST where each of the five laboratories tested ten blind samples: MICs of four ionophores (NAR, SAL, LAS and MON) for ten strains isolated from poultry originating from Lab 3. Putative NAR/SAL resistant strains are indicated in bold. Lab 1=OSR, Lab 2=NVI, Lab 3=WFSR, Lab 4=PIWet and Lab 5=ANSES.*

***Table S4****. Single MIC distributions generated from five different laboratories (Lab 1=OSR, Lab 2= NVI, Lab 3=WFSR, Lab 4=PIWet, and Lab 5=ANSES) for all* E. faecium *against all four ionophores (NAR, SAL, LAS and MON). Left side table comprises WT (narAB-negative) strains only, right side table includes narAB-positive strains (indicated in red for NAR and SAL MICs).*
